# Development, validation and application of a novel HPLC-MS/MS method for the quantification of atorvastatin, bisoprolol and clopidogrel in a large cardiovascular patient cohort

**DOI:** 10.1016/j.jpba.2018.06.062

**Published:** 2018-09-10

**Authors:** Richard Myles Turner, Vanessa Fontana, Mark Bayliss, Sarah Whalley, Anahi Santoyo Castelazo, Munir Pirmohamed

**Affiliations:** a The Wolfson Centre for Personalised Medicine, Institute of Translational Medicine, University of Liverpool, Liverpool, L69 3GL, UK; b Department of Microbiology, Southmead Hospital, Westbury-on-Trym, Bristol, BS10 5NB, UK; c Centre for Drug Safety Science, University of Liverpool, Liverpool, L69 3GE, UK

**Keywords:** Liquid chromatography-mass spectrometry, Assay validation, Atorvastatin, Bisoprolol, Clopidogrel

## Abstract

Cardiovascular disease is a leading cause of morbidity, mortality, and healthcare expenditure worldwide. Importantly, there is interindividual variation in response to cardiovascular medications, leading to variable efficacy and adverse events. Therefore a rapid, selective, sensitive and reproducible multi-analyte HPLC-MS/MS assay for the quantification in human plasma of atorvastatin, its major metabolites 2-hydroxyatorvastatin, atorvastatin lactone and 2-hydroxyatorvastatin lactone, plus bisoprolol and clopidogrel-carboxylic acid has been developed, fully validated, and applied to a large patient study. Fifty microliter plasma samples were extracted with a simple protein precipitation procedure involving acetonitrile with acetic acid (0.1%, *v/v*). Chromatographic separation was via a 2.7 μm Halo C18 (50 × 2.1 mm ID, 90 Å) column and gradient elution at a flow rate of 500 μL/min consisting of a mobile phase of water (A) and acetonitrile (B), each containing 0.1% formic acid (*v/v*), over a 6.0 min run time. The six analytes and their corresponding six deuterated internal standards underwent positive ion electrospray ionisation and were detected with multiple reaction monitoring. The developed method was fully validated with acceptable selectivity, carryover, dilution integrity, and within-run and between-run accuracy and precision. Mean extraction recovery for the analytes was 92.7–108.5%, and internal standard-normalised matrix effects had acceptable precision (coefficients of variation 2.2–12.3%). Moreover, all analytes were stable under the tested conditions. Atorvastatin lactone to acid interconversion was assessed and recommendations for its minimisation are made. The validated assay was successfully applied to analyse 1279 samples from 1024 patients recruited to a cardiovascular secondary prevention prospective study.

## Highlights

• Cardiovascular disease is a leading cause of morbidity and mortality.
• There is notable interindividual variation in response to cardiovascular drugs.
• An HPLC-MS/MS assay to quantify atorvastatin, bisoprolol and clopidogrel has been developed.
• The assay has been applied to 1279 plasma samples from 1024 patients.

## 1 Introduction

Cardiovascular disease (CVD) is a leading cause of morbidity and is responsible for ∼27% of all deaths worldwide [1]. The pathogenesis of CVD is complex and multifaceted [2,3]. Different drug classes are therefore frequently prescribed together for the secondary prevention of CVD, including: 3-hydroxy-3-methyl-glutaryl-coenzyme A reductase (HMGCR) inhibitors (statins, e.g. atorvastatin (ATV)), beta-adrenergic receptor antagonists (beta blockers, e.g. bisoprolol (BSP)) and antiplatelet drugs (e.g. clopidogrel (CLP)). It is axiomatic that drug effectiveness and toxicity are related to systemic drug concentrations to a certain extent, which can vary between individuals due to clinical (e.g. age, comorbidities), environmental (e.g. drug-drug and drug-food interactions) and genetic factors [4]. Therefore, there is a need to develop straightforward quantitative multi-drug assays and apply them to large patient cohorts to further parse the pharmacokinetic determinants of cardiovascular drug exposure, to determine the strengths of association between dose, exposure and outcome, and to potentially guide cardiovascular dosing in prospective studies and future clinical care.

Most of the available assays of human plasma or serum are limited to one cardiovascular drug [5,6] or therapeutic class [7,8]. One assay has been developed for qualitative detection of 34 cardiovascular drugs [9], and another for detecting 78 cardiovascular drugs, of which 55 compounds had appropriate accuracy and precision for quantitative determination [10]. Nevertheless, full validation is not undertaken with assays of this size, they do not employ analyte-specific deuterated internal standards (ISs) and extraction requires a potentially time consuming evaporation stage. Importantly, these assays have only been applied to modest numbers of samples from patients - for example 294 [9] and 13 [10].

**Fig. 1:**
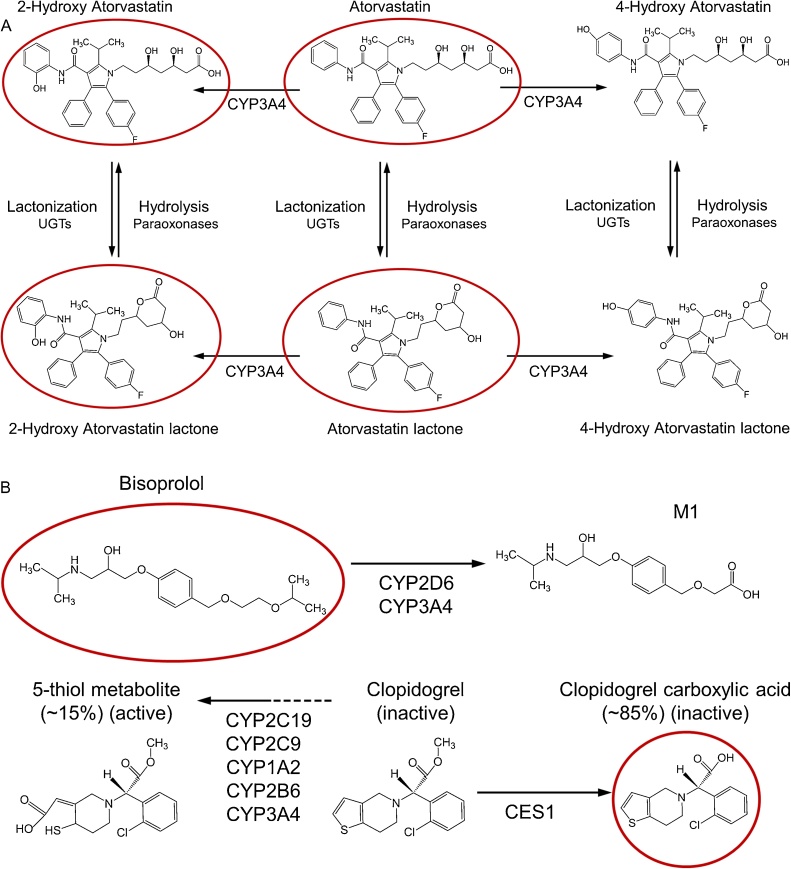
Chemical structures and relevant metabolism of analytes. Abbreviations: CES1 = carboxylesterase 1; CYP = cytochrome P450; UGTs = uridine 5'-diphospho-glucuronosyltransferases. The red rings denote the analytes quantified in this assay: parent ATV, its major hydroxylated metabolite (2-OH ATV), their corresponding lactones (ATV L and 2-OH ATV L), BSP, and the major CLP metabolite, CLP–CA. A: ATV and its hydroxylated acid metabolites, 2-OH ATV and 4-OH ATV, actively inhibit HMGCR to reduce circulating low-density lipoprotein cholesterol levels, although 2-OH ATV is the more abundant active metabolite [6]. The lactone metabolites are produced from the acid forms of ATV by UGTs, do not inhibit HMGCR, but are implicated in statin-induced muscle toxicity [[11], [12], [13]]. B: BSP is a cardioselective beta-1 adrenergic receptor antagonist racemate; ∼50% is renally excreted unchanged and ∼50% undergoes hepatic metabolism into labile or inactive metabolites prior to predominant renal excretion [14,15]. The inactive M1 metabolite of BSP is the most abundant BSP metabolite in human urine [15]. The active metabolite of CLP irreversibly inhibits platelet P2Y_12_ receptors, but is highly labile. Nevertheless, ∼85% of administered CLP is metabolised to the readily quantifiable inactive circulating metabolite, CLP-CA, which has been correlated with platelet inhibition indices [18], CLP non-adherence and variable metabolism [18,19].

The aim of this paper is to describe a newly developed, sensitive, precise, accurate and reproducible high performance liquid chromatography tandem mass spectrometry (HPLC-MS/MS) assay for the simultaneous quantification of ATV, BSP and CLP analytes in human plasma, and to apply this assay to one of the largest sparse pharmacokinetic studies reported to date. The assay identifies and quantifies six clinically relevant analytes from the three mechanistically distinct drugs: ATV, 2-hydroxy (2-OH) ATV, ATV lactone (ATV L), 2-OH ATV L, BSP, and CLP-carboxylic acid (CLP-CA) (Fig. 1). Briefly, ATV and its major hydroxylated acid metabolite, 2-OH ATV, actively inhibit HMGCR and lower circulating low-density lipoprotein cholesterol [6], whilst statin lactone metabolites are implicated in statin-induced muscle toxicity [[11], [12], [13]]. BSP is a cardioselective beta-1 adrenergic receptor antagonist administered as a racemate. Approximately 50% of BSP is renally excreted unchanged and ∼50% undergoes hepatic metabolism into labile or inactive metabolites that are excreted mainly through the kidneys, with the M1 metabolite being the most abundant in human urine [14,15]. CLP is an antiplatelet prodrug. Approximately 15% is rapidly metabolised to a highly unstable active 5-thiol metabolite that requires addition of a preservative immediately after blood collection for stabilisation [16,17], which precludes its accurate quantification in routinely collected samples. However, ∼85% of administered CLP is metabolised to the readily quantifiable inactive circulating metabolite, CLP-CA, which has been correlated with platelet inhibition indices [18], CLP non-adherence and variable metabolism [18,19].

## 2 Materials and methods

### 2.1 Chemicals and reagents

The following chemicals were purchased from Toronto Research Chemicals (Toronto, Canada): ATV calcium, 2-OH ATV calcium, ATV L, 2-OH ATV L, BSP hemifumarate, CLP-CA hydrochloride, and their respective deuterated ISs – ATV-d5 sodium, 2-OH ATV-d5 disodium, ATV-d5 L, 2-OH ATV-d5 L, BSP-d5 and CLP-d4 CA. Acetic acid, dimethyl sulfoxide (DMSO) and formic acid were obtained from Sigma-Aldrich (St. Louis, MO, USA). Acetonitrile, methanol and water were obtained from Fisher Scientific (Loughborough, UK). Pooled gender unfiltered human healthy volunteer plasma in K3 ethylenediaminetetraacetic acid (EDTA) was purchased from Sera Laboratories International (Seralab, West Sussex, UK). Unless stated otherwise, all reagents were HPLC-grade.

### 2.2 Preparation of solutions, standards and quality controls

Stock solutions were prepared by dissolving each chemical separately in DMSO (ATV, 2-OH ATV, ATV L, 2-OH ATV L, ATV-d5 L, 2-OH ATV-d5 L) or methanol (BSP, CLP-CA, ATV-d5, 2-OH ATV-d5, BSP-d5, CLP-d4 CA) and stored at -20 °C. Two sets of working solutions were prepared: a composite set consisting of ATV, 2-OH ATV, BSP and CLP-CA together in 25:75 acetonitrile-water (*v/v*); and a lactone set of ATV L with 2-OH ATV L in 100% acetonitrile. Both sets of working solutions consisted of a solution for each calibration standard, and a separately prepared solution for each quality control (QC), which were all prepared at 10x the required concentration in individual glass vials. Ten calibration standards were prepared in blank human EDTA-plasma for all analytes except ATV L, which required 8 standards, at the following concentrations: 0.5, 1.0, 1.5, 3.0, 10.0, 25.0, 50.0, 80.0, 100.0 and 125.0 ng/mL for ATV, 2-OH ATV, 2-OH ATV L and BSP; 1.2, 2.4, 8.0, 20.0, 40.0, 64.0, 80.0 and 100.0 ng/mL for ATV L and; 15, 30, 45, 90, 300, 750, 1500, 2400, 3000 and 3750 ng/mL for CLP-CA. The concentrations of the low, medium and high QCs in blank human EDTA-plasma were: 1.5, 45.0 and 95.0 ng/mL for ATV, 2-OH ATV, 2-OH ATV L and BSP; 3.6, 36.0 and 76.0 for ATV L and; 45, 1350 and 2850 ng/mL for CLP-CA. An intermediate IS working solution containing all six deuterated ISs in 100% acetonitrile was prepared from stock and stored at 4 °C. On each day of analysis, an aliquot of the IS intermediate solution was diluted 10x in 25:75 acetonitrile-water to produce the IS working solution (25 ng/mL for ATV-d5, 2-OH ATV-d5 and BSP-d5; 55 ng/mL for ATV-d5 L, 2-OH ATV-d5 L; and 750 ng/mL for CLP-d4 CA). All working solutions were stored at 4 °C, and kept on ice during use. Fresh calibration standards and QCs were prepared on each day of analysis during both validation and patient sample runs.

### 2.3 Sample extraction

Fifty microliters of plasma were transferred into a 96-well plate and 20 μL IS working solution was added. The samples were gently agitated for 8 min at 750 rpm (Orbit^™^ P2 digital shaker, Labnet International, USA) before 180 μL of 100% acetonitrile containing 0.3% acetic acid (*v/v*) was added for protein precipitation. The plates were further agitated for 2 min, centrifuged for 10 min at 2038×*g* at ca. 4 °C, and 100 μL of the supernatant was transferred to a new 96-well plate to which 200 μL of water was added; the final concentration of acetic acid was 0.1% (*v/v*). The plate was centrifuged for 5 min (2038×*g*, ca. 4 °C), before transfer to the autosampler. Full calibration lines were injected at the beginning and end of each analytical run.

### 2.4 LC–MS*/MS conditions*

A Shimadzu Nexera X2 modular system (Kyoto, Japan) was coupled to a Sciex triple quadrupole 6500 QTRAP mass spectrometer (AB Sciex, Warrington, UK). The Shimadzu system comprised a SIL-30AC autosampler, two LC-30AD pumps, a CTO-20 A column oven and a CBM-20 A controller. The autosampler injected a 5 μL aliquot of each sample into the system. The autosampler and oven temperatures were 4 °C and 40 °C, respectively. HPLC separation of analytes and ISs was accomplished using a 2.7 μm Halo C18 column (50 x 2.1 mm ID, 90 Å, Hichrom Limited, Reading, UK, part number: 92812-402) and gradient separation. Mobile phase A consisted of water with 0.1% *v/v* formic acid, and mobile phase B was acetonitrile with 0.1% *v/v* formic acid. The flow-rate was 500 μL/min, total run time was 6.00 min, and the gradient parameters were: 0.00–2.50 min 10% B, 2.51–3.75 min 50% B, 3.76–4.25 min 95% B, and 4.26–6.00 min 10% B. The MS analysis was carried out in the low mass setting with a Turbo V™ electrospray source operated in positive ionisation mode. An integrated 6-port Valco diverter valve was used to divert only the eluate containing the peaks of interest into the source. Detection and quantification were performed using multiple reaction monitoring (MRM, MS/MS). A dwell time of 9.0 msec per transition was used for all ATV analytes and corresponding ISs, and 7.0 msec per transition for BSP, CLP-CA and their ISs. Nitrogen, as nebuliser, heater, collision activated dissociation (CAD), and curtain gas (CUR), was supplied by a Genius 3031 nitrogen generator (Peak Scientific, Inchinnan, UK). The optimised gas settings were: CAD ‘medium’; CUR 25.0; GS1 (nebuliser) 50.0, and; GS2 (heater) 40.0. The other general, optimised, parameters were: turbo ionspray voltage 5500.0 V; entrance potential 10.0 V, and; source temperature 500 °C. Analyte and IS-specific MS parameters are listed in Table 1.

**Table 1:** MRM parameters.

**Chemical**	**MRM transition (Q1->Q3) (*m/z*)**	**Declustering potential (DP,V)**	**Collision energy (CE,V)**	**Collision cell exit potential (CXP,V)**	**Acquisition time (min)**
ATV	559.3 → 440.3	116	31	32	3.57
ATV-d5	564.1 → 445.1	46	29	38	3.57
2-OH ATV	575.3 → 250.2	111	57	50	3.48
2-OH ATV-d5	580.2 → 255.1	51	57	16	3.48
ATV L	541.3 → 276.2	50	55	24	3.77
ATV-d5 L	546.3 → 281.2	70	57	25	3.76
2-OH ATV L	557.3 → 276.2	60	57	24	3.66
2-OH ATV-d5 L	562.2 → 281.1	70	48	25	3.66
BSP	326.2 → 116.2	96	25	8	2.33
BSP-d5	331.0 → 121.1	96	23	10	2.33
CLP-CA	308.0 → 198.1	62	15	14	2.14
CLP-d4 CA	312.2 → 202.0	41	21	18	2.13

### 2.5 Method validation

The developed assay was validated for calibration curve performance, selectivity, carryover, accuracy, precision, matrix effects (MEs), stability and dilution integrity according to the European Medicines Agency guidelines [20].

#### 2.5.1 Selectivity

Selectivity was tested by analysing extracted blank plasma from six individuals, including one lipaemic and one haemolysed sample, to investigate interference at the retention time of each compound. Selectivity was accepted if the blank response of each individual sample was less than 20% of the mean (n = 6) lower limit of quantification (LLOQ) response for an analyte, and less than 5% of mean IS response for an IS.

#### 2.5.2 Carryover

Carryover into injected 25:75 acetonitrile-water, immediately following injection of the top calibration standard (upper limit of quantification, ULOQ), was assessed. Carryover was accepted if it was less than 20% of the LLOQ for an analyte, and less than 5% of IS response for an IS.

#### 2.5.3 Lower limit of quantification

The LLOQ for each analyte had a response at least 5x that of the mean blank response, and constituted the lowest calibration standard.

#### 2.5.4 Accuracy and precision

To evaluate the accuracy and precision of the method, four concentrations for each analyte (LLOQ and low, medium and high QCs) were analysed in six replicates per run. Three runs were carried out, and each run was conducted on a separate day by a different operator. Within run (n = 6) and between run (n = 18) accuracy and precision were determined. Accuracy was acceptable if the calculated concentration was within 15% of the nominal concentration for each QC, and within 20% at the LLOQ. Precision was acceptable if the coefficient of variation (CV) did not exceed 15% for each QC, and 20% for the LLOQ.

#### 2.5.5 Matrix effect and recovery

Matrix effect (ME) and extraction recovery (ER) were determined as described by Matuszewski et al [21]. Plasma from six individuals including one lipaemic and one haemolysed sample were tested at high and low QC concentrations. The MEs for each analyte and IS were determined separately in each sample by determining the ratio of the peak area in the post-extraction spiked plasma to the peak area in spiked 25:75 acetonitrile-water, and the IS-normalised ME for each analyte was calculated as the analyte ME/IS ME. A CV ≤ 15% for the IS-normalised ME from the six samples was acceptable. The ER was determined from the ratio of the peak areas of the pre-extraction spiked plasma to the post-extraction spiked plasma.

#### 2.5.6 Stability

The stability of the analytes in solution and in plasma (bench top, freeze-thaw, re-injection reproducibility and long term) was determined. Stock solution stability was assessed at three (ATV L, 2-OH ATV L) or six (ATV, 2-OH ATV, BSP, CLP-CA) months storage at ca. -20 °C, compared to fresh stock. Working solution stability was determined at four (composite analytes) or seven (lactone analytes) weeks storage at ca. 4 °C. Six replicates of spiked plasma at low and high QC concentrations were used to determine bench top (four hours on the work surface at room temperature), freeze-thaw (three cycles with at least 12 h freezer storage at ca. -80 °C between each cycle), and long term (three months at ca. -80 °C) stability, which were assessed against fresh calibration standards. The stability of extracts in the autosampler tray, set at 4 °C, was assessed by re-injecting calibration standards and QCs after they had been stored there for 24 h. In all stability studies, potential acid-lactone interconversion of the ATV analytes was assessed.

#### 2.5.7 Dilution integrity

For dilution integrity, six replicates of pooled plasma were spiked with composite or lactone analytes to high concentrations of: 800 ng/mL (ATV L), 1000 ng/mL (ATV, 2-OH ATV, 2-OH ATV L and BSP), and 30,000 ng/mL (CLP-CA). These high plasma samples were diluted 20-fold in plasma and tested with fresh calibration lines. Accuracy and precision (CV) of the diluted samples within 15% was considered acceptable.

### 2.6 Application to patient samples

The full validated method was applied to analyse steady-state concentrations in plasma samples collected during the Pharmacogenetics of Acute Coronary Syndrome (PhACS) study, which has been described previously [22]. Briefly, PhACS was a multicentre UK-based prospective cardiovascular observational study that ran from 2008 to 2013 and recruited 1470 patients hospitalised with a non-ST elevation acute coronary syndrome from 16 sites; participants were followed up for further cardiovascular events for at least 12 months. Blood samples were collected in EDTA-containing vacutainers at baseline, and at a median of one month (M1) and 12 months (M12) after discharge from index hospitalisation, transported at room temperature, centrifuged at 2600 *x g* for 20 min at room temperature, and stored at -80 °C. We included participants on one or more of ATV (80 mg or 40 mg daily at both index hospitalisation and M1), BSP (any dose) or CLP (75 mg daily) with adherence data for the drug(s) recorded at M1 (collected using the Brief Medication Questionnaire [23]) and an M1 plasma sample was available. In addition, a quarter of M1 analysed patients that had remained on ATV 80 mg daily, BSP (at the same dose between M1 and M12) or CLP at M12 with adherence data for the drug(s) recorded and plasma sample available were selected for analysis of their M12 drug levels. In total, twenty 96-well plates were analysed in 10 analytical runs (two plates/day). Each analytical run contained two set of calibration standards and four replicates for each QC (low, medium and high). The PhACS protocol was approved by the Liverpool (adult) research Ethics Committee, UK; site-specific approval was granted at all sites involved and local informed consent was obtained from all study subjects in accordance with the Declaration of Helsinki.

### 2.7 Data analysis

Raw data were processed using MultiQuant^™^ Version 1.6.2 (Sciex). The analyte/IS ratio of the peak area was calculated for the six analytes for calibration, QC and patient samples. Separate weighted least squares regression analyses were applied to the linear (1/x for ATV, 2-OH ATV, 1/x^2^ for BSP, ATV L, 2-OH ATV L) and quadratic (1/x for CLP-CA) calibration lines. In the patient study, concentrations below the LLOQ were excluded from further analysis. Patient drug concentrations are reported as the median (interquartile range, IQR) per dose. Pearson correlation analysis was performed to evaluate the relationship between the steady-state measurements at M1 and M12 using log_10_ transformed concentrations. The influence of sample storage duration (days) was assessed by linear regression of the M1 log_10_ transformed concentrations, with adjustment for dose carried out for all ATV analytes and BSP; the correlation coefficient (R^2^) relating specifically to the sample storage variable, its unstandardized regression coefficient (B), and corresponding p-value are reported. P-values are two-sided and p < 0.05 was considered statistically significant. Patient analyte concentrations were illustrated using R [24]; correlation and regression analyses were performed in IBM SPSS version 22.0 (IBM Corp, Armonk, NY, USA).

## 3 Results and discussion

### 3.1 *Optimisation of* LC–MS*/MS conditions*

**Fig. 2:**
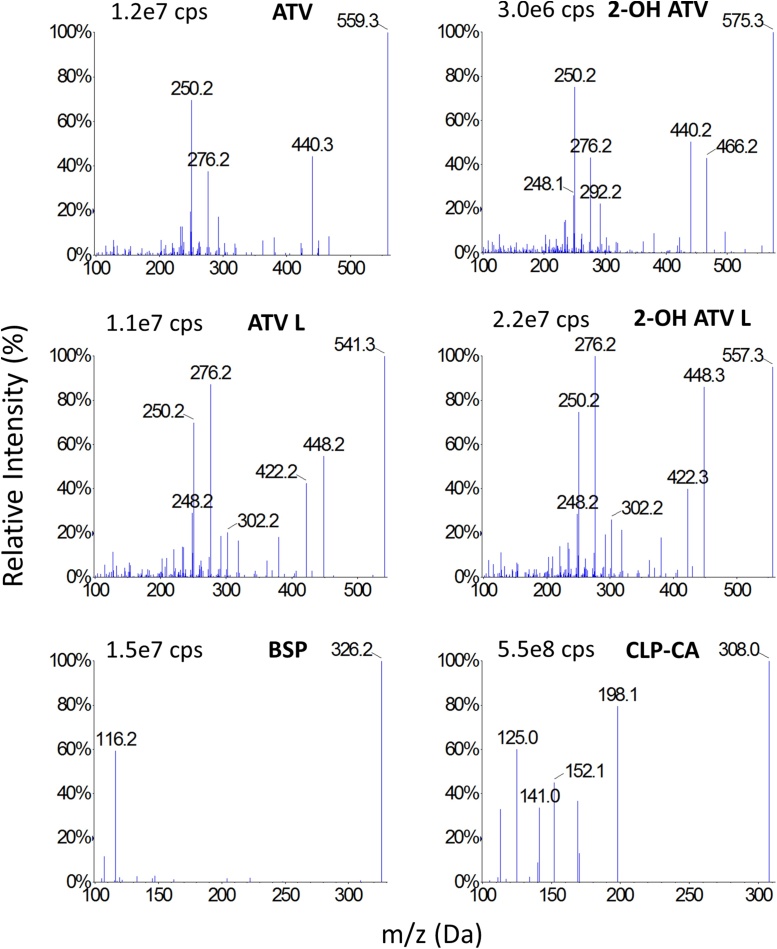
MS/MS spectra of analytes. Cps = counts per second. This figure shows the MS/MS precursor and product ion m/z spectra for each analyte. The cited cps refers to the highest peak (at 100%). The selected precursor and product ions for each analyte are: ATV 559.3 & 440.3; 2-OH ATV 575.3 & 250.2; ATV L 541.3 & 276.2; 2-OH ATV L 557.3 & 276.2; BSP 326.2 & 116.2; CLP-CA 308.0 & 198.1.

The 2.7 μm Halo C18 column was selected because it provided a symmetrical peak shape for all analytes and, in combination with the mobile phase and run time (6 min) used, it provided clear peak separation and both favourable intensity and selectivity. For instrument tuning and MS parameter optimisation, concentrated standard solutions of each analyte were infused separately using a syringe pump into the eluate from the column, prior to the MS. During tuning, the MS/MS parameters were systematically evaluated to optimise the response for each analyte and IS. The MS/MS spectra are shown in Fig. 2.

### 3.2 Optimisation of extraction procedure

**Fig. 3:**
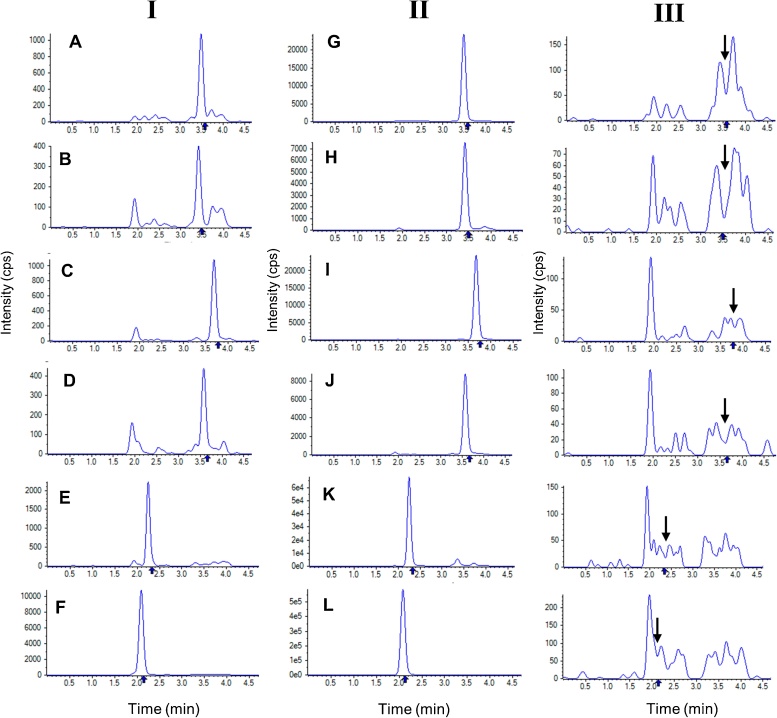
Representative MRM chromatograms of blank human plasma spiked with analytes at LLOQ (I), internal standard (II), or with no spiking (III). This figure shows representative multiple reaction monitoring chromatograms of: (I) blank human plasma spiked with analytes at the LLOQ; (II) blank human plasma spiked with internal standard, and; (III) blank human plasma with no spiking (i.e. double blank). The analyte retention times in the double blank extracts are denoted by the arrow. A = ATV; B = 2-OH ATV; C = ATV L; D = 2-OH ATV L; E = BSP; F = CLP-CA; G = ATV-d5; H = 2-OH ATV-d5; I = ATV-d5 L; J = 2-OH ATV-d5 L; K = BSP-d5; L = CLP-d4 CA.

A simple, rapid, reproducible and robust protein precipitation extraction method was desired so that minimal volumes of stored patient plasma samples could be analysed in support of a large sparse pharmacokinetic study. A deuterated IS was used for each analyte to aid selectivity and reproducibility. Assay recovery and reproducibility were then optimised through a two-step extraction procedure, where the IS solution (20 μL) was added to a spiked plasma sample (50 μL) and agitated, before the protein precipitation solvent (180 μL) was added and the mixture briefly re-agitated. The use of acetic acid within the acetonitrile precipitation solvent, to give a final concentration of 0.1% (*v/v*) (lowering plasma pH to 4–6), and maintaining the autosampler temperature at 4 °C were selected to reduce lactone to acid conversion of ATV lactone metabolites as previously reported [25,26]. The analyte, IS and double blank plasma representative chromatograms in Fig. 3 collectively demonstrate minimal endogenous interferences at the retention times of the six analytes and their deuterated IS. Overall, only 50 μL of sample was required, sample extraction within one hour was feasible, and 218 samples could be analysed over 24 h.

### 3.3 Method validation

The bioanalytical method described here met full validation criteria for calibration curve performance, selectivity, carryover, accuracy, precision, MEs, stability and dilution integrity (Table 2).

**Table 2:** Summary of validation results.

**Property**		******QC******	**ATV**	****2-OH ATV****	****ATV L****	****2-OH ATV L****	****BSP****	**CLP-CA**
Selectivity	Analyte (% LLOQ)	–	11.0	14.3	4.3	7.3	2.1	3.2
	Internal Standard (% IS)	–	0.71	0.90	0.28	1.35	0.09	0.08
Calibration curve performance	Correlation coefficient (*r*) 1	–	0.9995	0.9995	0.9982	0.9975	0.9983	0.9977
Dilution integrity	Accuracy (%)^2^	–	98.7	93.7	102.9	106.7	109.0	96.4
	Precision, CV (%)	–	11.7	6.2	3.4	4.9	14.0	3.3
Carryover	Analyte (% LLOQ)	–	6.2	3.7	2.7	10.9	1.1	5.2
	Internal Standard (% IS)	–	0.1	0.5	0.1	0.1	0.1	0.1
IS-normalised Matrix effect	Mean (%)^2^	Low	103.9	98.0	113.1	88.9	107.4	106.5
		High	112.2	98.4	114.7	102.8	106.3	100.9
	Precision, CV (%)	Low	12.3	12.2	11.6	8.8	7.0	8.8
		High	5.8	4.7	3.5	8.8	4.3	2.2
Recovery	Mean (%)^2^	Low	102.4	96.5	102.5	108.5	98.5	93.8
		High	99.1	98.8	98.2	100.6	97.4	92.7
	Precision, CV (%)	Low	13.3	13.9	8.9	5.4	11.0	12.4
		High	5.7	6.8	5.1	6.6	7.0	3.9
Within run accuracy and precision	Accuracy (%)^2^	LLOQ	95.0	97.0	90.3	107.6	92.8	101.5
		Low	104.5	102.2	98.8	108.6	102.8	102.7
		Medium	99.0	99.8	99.4	93.8	99.1	99.7
		High	96.1	94.3	99.0	97.8	96.0	95.0
	Precision, CV (%)	LLOQ	11.1	6.0	9.5	12.1	2.3	3.3
		Low	8.2	7.7	6.0	8.7	4.3	3.4
		Medium	1.6	2.2	2.3	4.2	2.0	2.3
		High	4.5	4.0	4.7	5.8	4.3	5.5
Between run accuracy and precision	Accuracy (%)^2^	LLOQ	95.3	96.1	100.0	100.5	94.4	97.0
		Low	103.7	101.5	98.2	101.2	102.8	101.1
		Medium	100.5	99.2	95.1	95.0	99.8	99.0
		High	99.0	97.3	98.0	99.8	99.0	98.0
	Precision, CV (%)	LLOQ	8.9	8.2	10.9	14.6	5.2	4.7
		Low	6.3	8.5	5.8	12.1	4.4	3.7
		Medium	2.6	4.1	4.6	5.7	2.6	3.5
		High	3.9	4.7	4.0	5.7	5.3	4.7
RT Benchtop stability (% of nominal concentration)		Low	114.3	107.7	100.3	105.6	98.5	107.7
		High	107.1	112.9	94.7	107.8	96.1	103.2
Freeze-thaw stability (% of nominal concentration)		Low	110.2	114.5	89.7	96.6	102.5	106.6
		High	103.6	102.4	88.2	96.0	100.9	99.2
Long term stability (% of nominal concentration)		Low	107.5	111.4	109.7	112.5	103.3	103.9
		High	104.4	106.0	106.4	103.2	104.5	99.8
Autosampler stability (% of nominal concentration)		Low	91.9	108.7	101.7	103.8	101.0	104.5
		High	103.9	101.1	97.4	99.0	99.7	103.9

Abbreviations: LLOQ = lower limit of quantification; IS = internal standard; QC = quality control; CV = coefficient of variation; RT = room temperature.

1 Average of 3 independent runs.

2 Accuracy is represented as % of nominal concentration.

The calibration curve was validated for all analytes over the following ranges: 0.5–125 ng/mL for ATV, 2-OH ATV, 2-OH ATV L and BSP, 1.2–100 ng/mL for ATV L, and 15-3,750 ng/mL for CLP-CA. The LLOQ was 0.5 ng/mL for ATV, 2-OH ATV, 2-OH ATV L and BSP, 1.2 ng/mL for ATV L and 15 ng/mL for CLP-CA. The within run and between run accuracy and precision of the LLOQ and QCs for all analytes were within the accepted range. Following a 20-fold dilution, for all analytes the accuracy was 93.7–109% and the CV 3.3–14%; therefore, dilution up to 20 times for patient samples higher than the ULOQ was acceptable. Analyte responses were stable in plasma on the benchtop (four hours, room temperature), after three freeze-thaw cycles, after three months of storage (at ca. -80 °C), and in the autosampler (24 h, set at 4 °C). Moreover, analyte responses in stored stock and working solutions were within 15% of the fresh solution responses, which was considered acceptable.

Lactonization of the acid form and hydrolysis of a lactone to the open-acid form of ATV are mediated by uridine 5′-diphospho-glucuronosyltransferases (UGTs) [27] and paraoxonases [28], respectively. ATV analyte acid-lactone interconversion has been investigated extensively before [26]. In keeping with these previous findings, the interconversion of ATV to ATV L, and 2-OH ATV to 2-OH ATV L, was uniformly negligible in our assay, with an increase in the proportion of lactone species of ≤0.3% in all stock, working solutions, and plasma-based stability studies. The lactone metabolites and particularly ATV L are more unstable [26]. However, this can be almost completely negated by lowering the temperature to 4 °C and/or lowering the pH during extraction with acetic acid [26]. In our assay, ATV L to ATV and 2-OH ATV L to 2-OH ATV conversion in stability studies was less than 5%, except for ATV L to ATV in plasma after three freeze-thaw cycles and after four hours at room temperature. Nevertheless, sample extraction within one hour is feasible and lactone to acid conversion after one hour in spiked plasma on the benchtop was <5%. Furthermore, lactone to acid conversion after four hours on the bench top in ice (ca. 4 °C) was <1.5%. Thus, we recommend sample extraction without delay if samples undergo extraction at room temperature, or extraction on ice if delays are expected. Minimising the number of freeze-thaw cycles, modestly lowering the pH during extraction and running the autosampler at ca. 4 °C are all recommended.

### 3.4 Assay application to large clinical study

**Fig. 4:**
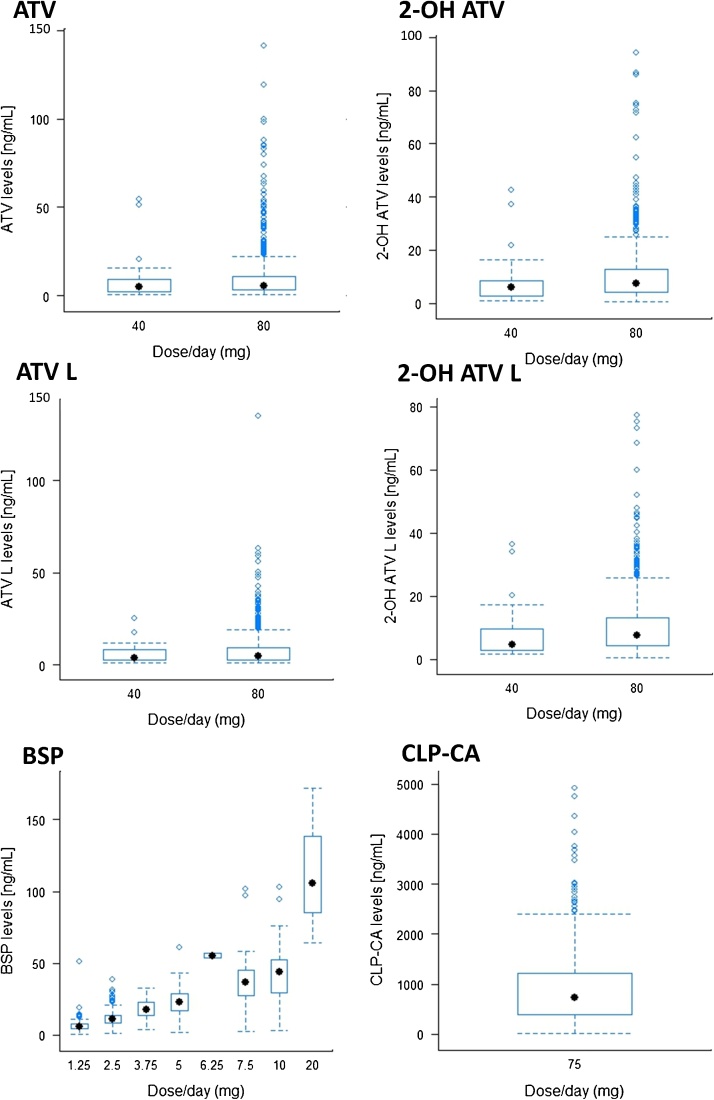
Drug plasma levels obtained from PhACS patients.

The developed HPLC-MS/MS method was successfully applied to measure analyte concentrations in 1279 samples from 1024 patients (1024 samples at M1 and 255 at M12). Included samples had undergone a maximum of one freeze-thaw since first storage. The number of patients that met the M1 inclusion criteria for ATV, BSP and CLP-CA were 718, 736 and 811, respectively. At M12, the number of eligible samples analysed for ATV, BSP and CLP-CA were 173, 182 and 205 respectively. Median (IQR) analyte concentrations are listed in Table 3 and represented graphically in Fig. 4.

**Table 3:** Summary of analyte concentrations for each dose in plasma samples.

Analyte	M1, n	Dose (mg)	Samples at M1 above LLOQ, n (%)^1^	Conc. (ng/mL) Median (IQR)	M12, n	Samples at M12 above LLOQ, n (%)^1^	Conc. (ng/mL) Median (IQR)	M1 & M12 correlation	
								Pearson correlation	p-value
**ATV**	718	40	43 (97.7)	4.86 (1.92-9.53)	173	–	–	–	–
		80	631 (93.6)	5.67 (3.19-10.98)		157 (90.7)	4.97 (2.81-8.48)	0.346	1.40 × 10^−5^
**2-OH ATV**		40	42 (95.5)	5.85 (2.80-8.81)		–	–	–	–
		80	640 (95.0)	7.49 (4.26-12.76)		163 (94.2)	7.48 (3.86 – 11.42)	0.294	1.76 × 10^−4^
**ATV L**		40	31 (70.5)	3.98 (2.51-8.90)		–	–	–	–
		80	569 (84.4)	4.94 (2.87-9.41)		131 (75.7)	4.33 (2.71-7.46)	0.316	3.88 × 10^−4^
**2-OH ATV L**		40	42 (95.5)	4.64 (2.74-9.73)		–	–	–	–
		80	644 (95.5)	7.49 (4.39-13.22)		165 (95.4)	6.63(4.07-9.86)	0.268	5.94 × 10^−4^
**BSP**	736	1.25	140 (99.3)	5.67 (4.31-7.63)	182	26 (96.3)	5.50 (4.28-7.05)	0.640	4.37 × 10^−22^
		2.5	316 (98.8)	10.83 (8.24-14.05)		83 (100.0)	10.27 (6.78-13.67)		
		3.75	21 (100.0)	17.50 (12.53-23.05)		6 (100.0)	13.34 (10.13-18.27)		
		5	167 (96.0)	23.14 (17.08-28.86)		49 (98.0)	20.32 (15.50-30.68		
		6.25	2 (100.0)	55.34^2^		1 (100.0)	58.7^4^		
		7.5	24 (96.0)	36.74 (27.35-45.67)		4 (100.0)	32.38 (12.70-51.56)		
		10	49 (98.0)	43.93 (28.06-52.73)		10 (90.9)	53.37 (37.17 – 68.51)		
		20	3 (100.0)	105.49^3^		1 (100.0)	167.2^4^		
**CLP-CA**	811	75	792 (97.7)	736.94 (392.13-1211.11)	205	195 (95.12)	(737.58 (414.61-1124.49)	0.255	3.34 × 10^−4^

Concentrations below the LLOQ were excluded from the median (IQR) concentration calculations and M1 to M12 correlation analysis. Pearson correlation analysis was performed on log_10_ transformed concentrations. ^1^ = the LLOQ was 0.5 ng/mL for ATV, 2-OH ATV, 2-OH ATV L and BSP, 1.2 ng/mL for ATV L, and 15 ng/mL for CLP-CA; ^2^ = n = 3 samples; ^3^ = n = 3 samples; ^4^ = n = 1 sample.

Overall, we found that at least 93% of samples have analyte levels above the LLOQ (M1 and M12) in this ‘real world’ cohort. Specifically, the proportion of samples above the LLOQ at M1 were 93.9% for AVT, 95.0% for 2-OH AVT, 83.6% for ATV L, 95.5% for 2-OH ATV L, 98.1% for BSP and 97.7% for CLP-CA. The proportion of samples with ATV L levels above the LLOQ was lower than for the other ATV analytes, because of its higher LLOQ (1.2 ng/mL). However, if necessary the LLOQ of ATV L could be relaxed to 0.5 ng/mL for consistency with the other ATV analytes, which had an accuracy and precision (CV) of 88.8% and 28.2%, respectively, to yield an equivalent number of valid samples (n = 655). Some, but not all, of the values below the LLOQ appear related to patient self-reported non-adherence. The highest measured concentrations were: 141 ng/mL (ATV), 94.3 ng/mL (2-OH ATV), 135 ng/mL (ATV L), 77.3 ng/mL (2-OH ATV L), 172 ng/mL (BSP), and 4921 ng/mL (CLP-CA).

The M1 plasma samples had been stored long term for a mean of 2317 days prior to analysis. Thus, we were unable to perform a long term stability assessment that spanned this duration, and so we investigated whether sample duration (in days) was associated with measured analyte levels. Univariate linear regression analysis showed no significant associations for ATV, BSP or CLP-CA levels. However, sample storage duration was associated with reduced 2-OH ATV (B = 1.38 × 10^−4^, R^2^ = 2.5%, p = 3.10e^-05^), and increased ATV L (B = 1.84 × 10^−4^, R^2^ = 4.4%, p = 1.83 × 10^-7^) and 2-OH ATV L (B = 1.01 × 10^−4^, R^2^ = 1.3%, p = 0.0027) responses. Whilst the influence of sample duration was strongest for ATV L, it still accounted for less than 5% of observed variability in observed concentrations, which was considered acceptable.

Interestingly, there were significant correlations for all analytes between the M1 and M12 steady-state measurements (Table 3). Overall, these results demonstrate the utility of the assay for analysis of large sample sizes; further analyses of the data are ongoing to relate clinical and pharmacogenomic factors to these analyte levels and subsequent cardiovascular events.

## 4 Conclusion

We have developed and validated a novel, sensitive, reproducible and robust assay for the quantification in human plasma of six analytes highly relevant to CVD, and applied this assay, to the best of our knowledge, to one of the largest sparse pharmacokinetic studies undertaken to date. The assay requires a small sample volume, incorporates a simple extraction procedure and rapidly separates analytes; thus, it is amenable for use in large studies. Millions of patients are regularly prescribed concomitant ATV, BSP and CLP. However, in order to advance precision medicine, it is necessary to further parse and act on the factors associated with interindividual drug response variability; simple and easy-to-use multi-analyte assays, like the one reported here, will be pivotal to this effort.

## Funding/support

RMT was supported by the NW England MRC Scheme in Clinical Pharmacology and Therapeutics. VF was supported by the Newton Fund and the UK Academy of Medical Sciences. MP is supported through the MRC Centre for Drug Safety Science. The clinical study was funded as part of the NHS Chair of Pharmacogenetics, Department of Health.

## Conflict of Interest

All authors declare no conflicts of interest.
